# Trypanosomatid protein phosphatases

**DOI:** 10.1016/j.molbiopara.2010.05.017

**Published:** 2010-10

**Authors:** Balázs Szöör

**Affiliations:** Centre for Immunity, Infection and Evolution, Institute of Immunology and Infection Research, School of Biological Sciences, University of Edinburgh, King's Building, West Mains Road, Edinburgh EH9 3JT, UK

**Keywords:** Protein phosphatases, Trypanosomatids, *Trypanosoma*, *Leishmania*, Signal transduction

## Abstract

Protein phosphorylation is one of the most important post-translational modifications regulating various signaling processes in all known living organisms. In the cell, protein phosphatases and protein kinases play a dynamic antagonistic role, controlling the phosphorylation state of tyrosine (Tyr), serine (Ser) and threonine (Thr) side chains of proteins. The reversible phosphorylation modulates protein function, through initiating conformational changes, which influences protein complex formation, alteration of enzyme activity and changes in protein stability and subcellular localization. These molecular changes affect signaling cascades regulating the cell cycle, differentiation, cell–cell and cell–substrate interactions, cell motility, the immune response, ion-channel and transporter activities, gene transcription, mRNA translation, and basic metabolism. In addition to these processes, in unicellular parasites, like *Trypanosoma brucei*, *Trypanosoma cruzi* and *Leishmania* spp., additional signaling pathways have evolved to enable the survival of parasites in the changing environment of the vector and host organism. In recent years the genome of five trypanosomatid genomes have been sequenced and annotated allowing complete definition of the composition of the trypanosomatid phosphatomes. The very diverse environments involved in the different stages of the kinetoplastids’ life cycle might have played a role to develop a set of trypanosomatid-specific phosphatases in addition to orthologues of many higher eukaryote protein phosphatases present in the kinetoplastid phosphatomes. In spite of their well-described phosphatomes, few trypanosomatid protein phosphatases have been characterized and studied *in vivo*. The aim of this review is to give an up to date scope of the research, which has been carried out on trypanosomatid protein phosphatases.

## Graphical abstract

Classification of kinetoplastid serine/threonine specific and protein tyrosine phosphatases based on sequence similarity. The groups absent from kinetoplastids are in white boxes. In spite of well-described kinetoplastid phosphatomes, few kinetoplastid protein phosphatases have been characterized and studied *in vivo*.

## 1 Introduction

### 1.1 Trypanosomatids and diseases

The members of the Kinetoplastida are flagellated unicellular organisms, including extra and intracellular parasites responsible for severe diseases in humans and animals, as well as various free-living forms found in soil and aquatic environments. In this review I will focus on the three most important human pathogens *Trypanosoma brucei*, *Trypanosoma cruzi*, and *Leishmania* and their protein phosphatases, since these have available genome sequences and are the most experimentally characterized.

**Fig. 1:**
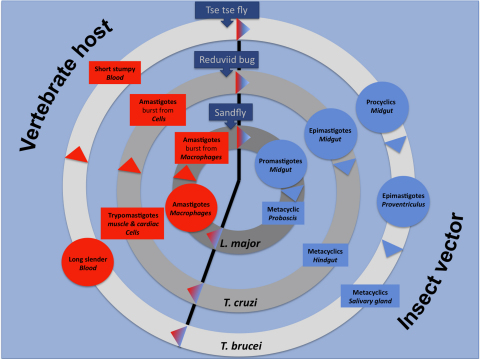
Combined life cycles of the three Trypanosomatid species in their vertebrate host and their transmitting vectors (tsetse fly (*T. brucei*), reduviid bug (*T. cruzi*) and sandfly (*L. major*)). The life cycle stages shown for *T. brucei* in the vertebrate host's blood: dividing long slender form, and the non-dividing short stumpy form, awaiting for transmission. In the tsetse fly vector: the proliferative procyclics in the midgut and epimastigotes in proventriculus and the non-dividing metacyclics, ready for transmission, in the salivary gland. *T. cruzi*: the trypomastigotes invade various cells (including cardiac and muscle cells) and transforms into amastigotes which either burst from the infected cells and await for transmission, or infect new cells. In the reduviid bug the proliferating epimastigotes are found in the midgut and the non-dividing metacyclics colonizing the hindgut. *L. major*: the proliferative amastigotes invade macrophages, after filling up the cells burst from the infected cells and await for transmission. In sandfly the proliferating promastigotes are colonizing the midgut and the non-dividing metacyclics can be found in the proboscis. The proliferative life cycle stages are circled, and the non-dividing forms were boxed in each life cycles. The life cycle stages in the insect vectors are highlighted in blue, and the vertebrate host stages are in red. Arrowheads with gradient red to blue colour show transmission of the parasites from the mammals to the insect vector, and arrowheads with blue to red gradient mark the vector to vertebrate host passage. Based on Ref. [1].

*T. brucei* and its subspecies are transmitted by tsetse flies (Fig. 1 modified from Ref. [1]) and cause human African trypanosomiasis (HAT) and nagana in cattle. African sleeping sickness threatens over 50 million people in 36 sub-Saharan African countries, and has an enormous effect also on the livestock and economic development of affected areas. Unlike sleeping sickness, Chagas disease is caused by the intracellular pathogen, *T. cruzi*, transmitted by blood feeding triatomid insects (Fig. 1 modified from Ref. [1]). The disease is endemic throughout Latin America, where the estimated number of cases is around 8–10 million. Pathogens belonging to *Leishmania* taxon, are intracellular and transmitted by sandflies (Fig. 1 modified form). They cause various diseases ranging from self-healing cutaneous leishmaniasis to severe (and lethal if untreated) visceral leishmaniasis (also known as kala-azar), a common infectious disease in Southern Europe, Africa, Asia and the Americas, killing thousands and debilitating millions of people each year.

The combined number of people infected by kinetoplastid pathogens is estimated to be over 20 million, resulting in various health problems and more than 100,000 deaths each year. With half a billion of people at risk, mostly in the tropical and subtropical areas of the world, these parasites represent an important global health problem with associated significant economic burden [2].

### 1.2 Bioinformatic analysis of TriTryp genomes and proteomes

The genomes of three kinetoplastid parasites *T. brucei* [3] *T. cruzi* [4] and *Leishmania major* [5] have been published, followed by a study of comparative genomics study giving insight into the evolutionary similarities and differences between the *L. infantum* and *L. braziliensis* genomes [6]. These studies enabled the assembly of the TriTryp kinome [7] and phosphatome [8] providing a compilation of kinases and phosphatases encoded in the respective parasite genomes.

Protein phosphatases remove phosphate groups from various phosphorylated amino acids. The most predominant phosphorylation sites in eukaryotic cells are detected on serine, threonine and tyrosine residues. The first phosphoproteome analysis of kinetoplastids [9] identified 491 phosphoproteins in the bloodstream form of *T. brucei*, which means 5.5% of the proteins were phosphorylated in this life cycle stage, although this is very likely a small fraction of the phosphorylated proteins found in an intact cell. The majority of the identified proteins were phosphorylated on Ser or Thr (75 and 21.5% respectively) and only 3.5% were Tyr phosphorylated, thereby showing a similar (although slightly reduced percentage of tyrosine-phosphorylated proteins) phosphorylation pattern described in bacteria (69/22/9% Ser/Thr/Tyr) [10]. In vertebrates (*HeLa* cells) an even lower percentage of tyrosine-phosphorylated proteins have been described (86.4/11.8/1.8%) [11] compared to the aforementioned unicellular organisms.

### 1.3 Classification of protein phosphatases

**Fig. 2:**
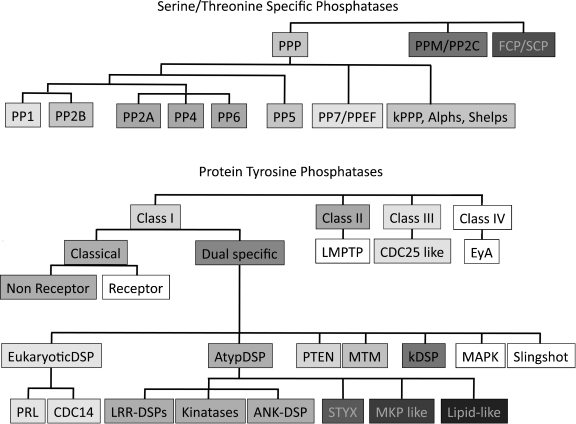
Classification of serine/threonine specific (A) and protein tyrosine phosphatases (B). Protein phosphatases were classified on the basis of sequence similarity. The shades of boxes denote protein phosphatases belonging the same subfamilies, the phosphatase groups absent from Trypanosomatidae have been boxed in white.

Protein phosphatases can be classified into four major groups based on catalytic signature motifs and substrate preferences: phosphoprotein phosphatases (PPPs), metallo-dependent protein phosphatases (PPMs), aspartate-based phosphatases with a DxDxT/V motif (the members of these three groups are Ser/Thr specific phosphatases) and the distinct group of protein tyrosine phosphatases (PTPs) (Fig. 2A and B and Tables 1 and 2) [12].

## 2 Serine/threonine specific phosphatases (STPs)

In eukaryotes the majority of proteins (96–99%) are phosphorylated on Ser and Thr residues. STPs are responsible for the majority of dephosphorylation events of these residues in the cell and classified into three major families based on sequence homology, metal ion dependence, sensitivity for various inhibitors and catalysis based mechanisms [13]. The three families of the STPs are: (i) PPPs, (ii) PPM/PP2C families and (iii) DxDx(S/T) phosphatases (Fig. 2A and Table 2). The genes encoding the PPPs and PPMs show very low homology to each other, despite a very similar 3D structure surrounding the catalytic centre, and form evolutionary distinct unrelated groups of the Ser/Thr specific phosphatases [14]. Also, in contrast to members of PPM family, which do not have regulatory subunits, the enzyme activity, substrate specificity, subcellular localization of PPPs are regulated by various regulatory subunits [15,16].

For both PPP and PPM, metal ions play an important role in catalysis through the activation of a water molecule for the dephosphorylation reaction. The PPP family is subdivided into type1 (PP1), type2a (PP2A) and the closely related PP4 and PP6, type PP2B (or PP3), PP5 and PP7 subgroups (Fig. 2A) [15]. The members of the PPM family are Mn^2+^/Mg^2+^ dependent enzymes, such as PP2C and pyruvate dehydrogenase phosphatase. The enzymes belonging the PPP and PPM family dephosphorylate the majority of phospho-serine and phospho-threonine residues.

The third, most recently classified group of STPs contains the aspartate-based phosphatases represented by FCP/SCP (TFIIF-associating component of RNA polymerase II C-terminal domain CTD phosphatase/small CTD phosphatase), and haloacid dehalogenases (HAD) with phosphatase activity [17,18].

In contrast, to the PPP and PPM groups, FCPs/SCPs use an aspartate-based (DxDxT/V) catalytic core to dephosphorylate phospho Ser/Thr. The conserved structural core of FCP/SCP is the FCP homology (FCPH) domain. In addition to this in FCPs, a BRCT (BRCA1 C-terminal domain like) domain is present C-terminal to the FCPH domain, which is absent from SCPs [17,19].

### 2.1 STP inhibitors

Through decades of phosphatase research an array of specific STP inhibitors have been identified first to classify, then to investigate the physiological role of STPs [20].

In mammalian cells, the cell permeable okadaic acid (OA) is a potent PP2A inhibitor (the 50% inhibitory concentration, IC_50_: 2 nM), whereas higher concentrations are necessary to inhibit PP1 (IC_50_: 60–200 nM) or PP2B (IC_50_: 10 mM) and PP2C is unaffected by this inhibitor. In contrast, calyculin A inhibits both PP1 and PP2A, but not PP2B or PP2C, with high potency (IC_50_: 0.5–1 nM). Tautomycin is a potent inhibitor of PP1 (IC_50_: 1 nM) and 10 times higher concentrations are necessary to inhibit PP2A (IC_50_: 10 nM), whereas PP2B is weakly inhibited and PP2C is unaffected by this compound [21].

### 2.2 Trypanosomatid STPs

#### 2.2.1 PP1

Although protein phosphatase activity was detected in *T. brucei* more than a quarter of a century ago [22] still little is known about the physiological role of protein phosphatases in kinetoplastids. The reason for this may be the complicated life cycles of the different kinetoplastids (Fig. 1 adapted from Ref. [1]), and the difficulties in culturing and genetic manipulation of the various lifeforms. In addition, the TriTryp phosphatome [8] identified a relatively high number of STPs (54/56/58 in *T. brucei*/*T. cruzi*/*L. major* respectively (Supplementary Table 1), compared to 39 in human (Table 1)), showing high similarity and very likely sharing overlapping roles, further complicating functional analysis of these enzymes.

Analysis of TriTryp genomes identified 8/7/8 (*T. brucei*/*T. cruzi*/*L. major*) PP1s of which 4 in *T. brucei*, 3 in *T. cruzi* and 5 in *L. major* were found in tandem gene arrays (highlighted in bold in Supplementary Table 1a). The reason for this unique allocation of phosphatase genes has not been investigated, but it may have evolved to regulate the differential expression of the different isoforms.

In the early nineties Erondu et al. cloned and characterized two PP1s (named PP1A (Tb927.8.7390) and B (Tb927.4.5030)) and one PP4 (Tb11.01.8740) (named PP2A) of *T. brucei* [23] (Table 3) and found a remarkable similarity to their mammalian counterparts, despite the early divergence of kinetoplastids from the main branch of eukaryotes [24]. Interestingly the PP1 genes were co-transcribed with the gene encoding the RNA polymerase II (RNAPII) largest subunit [25] and the authors suggested that the RNAPII could be a substrate for the PP1 in this parasite. Although in multicellular organisms RNAPII dephosphorylation is mediated by PP1 *in vitro*, and probably *in vivo*, too [26] (in addition to the activity of FCP1), to date there is no evidence that kinetoplastid PP1 is capable of the *in vivo* dephosphorylation of RNAPII.

In the early years of phosphatase research the only tool to identify the physiological role of the enzymes was the use of specific phosphatase inhibitors. The inhibitors, which were used previously to classify Ser/Thr specific phosphatases in mammalian cells, have also been used to study the role of STPs in kinetoplastids. When trypanosomes were treated with OA, the cells were defective in segregation of the organellar genome and cytokinesis but not in mitosis, suggesting that the role that protein phosphorylation plays in cell division may also include a role in the organellar cycle [27].

The available data indicate that these events are coordinated within the cell cycle [28], although little is still known about the specific molecules involved. Since treatment with OA overrides this coordination, the authors suggested that a protein phosphatase might function in the coupling of mitosis and the cell cycle of trypanosomes. In contrast to the effect of OA, the combined RNAi ablation of 7 PP1s and the PP2A catalytic subunits in procyclic forms, resulted in a slow growth phenotype [29] without causing any severe phenotype or increased number of multinucleated cells. The explanation for these conflicting reports may be the lack of total ablation of the protein phosphatase activities by RNAi, the different roles of STPs in different life cycle stages and/or a presence of another OA sensitive protein phosphatase in *T. brucei*. For example, inhibition of PP1 and PP2A activity by high concentrations of OA (1 μM) resulted in down-regulation of beta-tubulin mRNA gene expression in *T. b. rhodesiense* [30], although it is not clear the effect was mediated.

In *T. cruzi* [31], use of a range of PP1 and PP2A inhibitors (OA, tautomycin (TA) and calyculin A (CA)) demonstrated that the trypomastigotes treated with low concentrations (1 nM) of CA underwent differentiation, spontaneously to generate rounded amastigote form, and expressed all the markers observed after the normal differentiation process. This phenomenon was specific to the CA treated cells, suggesting that the CA-sensitive protein phosphatase activity, which was detected in the cytoskeletal fraction, may play a key role in the remodeling of cell shape, either by directly dephosphorylating cytoskeletal proteins, or indirectly, by dephosphorylating and activating kinases involved in cytoskeleton phosphorylation, as has been observed in mammalian cells [32]. Also it was noted that, in spite of the similar features of the pH and CA induced transformation of trypomastigotes into amastigote-like forms, different phosphorylation patterns were observed in each case, suggesting that multiple signals may be involved in the regulation of transformation.

Two PP1 isoforms (*Tc*PP1α (Tc00.1047053506201.70) and β (Tc00.1047053507671.39)) were identified in *T. cruzi* and the mRNAs were detected in both epimastigote and metacyclic parasites by Orr et al. [33] (Table 3). Calyculin A-treated epimastigotes underwent flagellar duplication and both kinetoplast and nuclear divisions but were incapable of successfully completing cytokinesis. These cells also lost their characteristic elongated, epimastigote phenotype and adopted a more rounded morphology. The authors suggested these PP1-like phosphatases are important for the completion of cell division and the maintenance of cell shape in *T. cruzi*.

##### 2.2.1.1 PP2B/calcineurin

Two distinct types of PP2B were identified in the TriTryp phosphatome, the first group clustered together with yeast and vertebrate enzymes, while the second group showed less similarity to other calcineurins [8]. An enzyme with Ca^2+^ dependent PP2B activity was partially purified from the cytosol of *Leishmania donovani* promastigotes (Table 3). This *Ld*PP2B exhibited similar properties to the calcineurins isolated from various species [34]. A novel homologue of PP2B from *T. cruzi* was also identified and characterized by Moreno et al. [35]. The *Tc*PP2B (Tc00.1047053508413.40) is expressed in all major developmental stages of *T. cruzi* and it is mainly localized in the cell nucleus (Table 3), in sharp contrast with the mammalian calcineurin A, which is mainly found in the cytoplasm and translocates to the nucleus after binding to its receptor [36]. Out of the four conserved domains typically present in all calcineurins [37], the *Tc*PP2B has only the catalytic and the calcineurin B binding domains and neither the calmodulin-binding, nor the auto-inhibitory domain can be identified. Interestingly, after the analysis of the kinetoplastid PP2B amino acid sequences the authors found that only the *L. major* calcineurin homologue contained all the four characteristic PP2B conserved domains described in other species [35].

##### 2.2.1.2 PP2A, PP4, PP6

Each TriTryp genome encode two PP2A isoforms, one of which is closely related to yeast and vertebrate counterparts, while the second is more distant from higher eukaryote PP2As. The *T. cruzi* member of the latter group was characterized in an attempt to investigate whether *Tc*PP1 or *Tc*PP2A (Tc00.1047053511021.10) were involved in the transformation of trypomastigotes into amastigotes [38]. In transformation assays at pH 5.0, even low concentrations (0.1 μM) of OA had a profound effect on the transformation of trypomastigotes while TA, a known PP1 inhibitor, only had moderate effect (at concentrations up to 10 μM), suggesting that it is the *Tc*PP2A-type enzymes that are involved in parasite transformation.

Kinetoplastids have only one isoform of PP4 and PP6, except *T. brucei*, which lacks the PP6 homologue (Table 1 and Supplementary Table 1d and e). The *Tb*PP4 was the first novel STP cloned from a kinetoplastid, although it was referred to as PP2A [23]. To date no physiological role is known for either PP4 or PP6 in kinetoplastids.

##### 2.2.1.3 PP5

The PP5s are characterized by their N-terminal tetratricopeptide repeat (TPR) domains, with a role in protein–protein interaction and in auto-inhibition [39]. There is one gene in each TriTryp genome encoding PP5 (Table 1 and Supplementary Fig. 1f), but only the *Tb*PP5 (Tb927.10.13670) has been characterized.

This molecule was found in the cytosolic/nuclear fraction of the cell by Chaudhuri [40]. All the invariant structural motifs (-GDXHG-, -GDXVXRG- and -GNH-) described in the members of PPP family [41] were present in *Tb*PP5, as well as in all the *T. brucei* STPs characterized to date [8]. The N terminus of *Tb*PP5 contained 3 TPR domains and its activity was stimulated by arachidonic acid as described for mammalian PP5 by Chen and Cohen [42].

The protein level was found to be slightly higher in procyclic forms compared to bloodstream forms (Table 3) and the transcript level decreased in cells transferred from the logarithmic phase growth to the stationary phase in culture. In procyclic cells, following 18 h starvation, the transcript level of *Tb*PP5 was reduced approx 3-fold, suggesting a role for this phosphatase in the active growth phase of the parasite. Through its TPR motifs, PP5 interacts with various stress-related proteins including Hsp90 in other eukaryotes [43]. In *T. brucei*, an essential Hsp90 homologue (*Tb*Hsp83) with reasonably high similarity to its vertebrate counterpart and very high ATPase activity was identified [44]. Recently, it was shown that *Tb*Hsp83 interacts with *Tb*PP5 *in vivo* and both *Tb*PP5 and *Tb*Hsp83 accumulate in the nucleus during proteotoxic stresses [45]. The authors showed that both in bloodstream and procyclic forms over-expressing *Tb*PP5 reduced, and ablation of *Tb*PP5 increased, the growth inhibitory effect of the specific Hsp90 inhibitor geldanamycin. This effect was more pronounced in bloodstream form compared to procyclics, suggesting that *Tb*PP5 may be involved in regulating Hsp90 function under stress, either increasing the chaperon function of *Tb*Hsp83 via stabilizing the *Tb*Hsp83–substrate complex or maintaining the dephosphorylated state of *Tb*Hsp83 [45].

##### 2.2.1.4 PP7/PPEF (protein phosphatases with EF-hand)

The PP7 group shows sequence similarity to PP5, but they are regulated by a calmodulin-binding domain at the N-terminal, and the Ca^2+^ binding EF-hand motifs at the C-terminal regions of the molecule.

There are two PP7 in *T. brucei* and *T. cruzi*, but only one gene was identified in *L. major* [46] (Table 1 and Supplementary Table 1g). In kinetoplastids the members of the PPEF family do not have a calmodulin-binding motif and their EF-hand motifs differ from the consensus. Because of the missing calmodulin-binding domain, and the modified EF-hand, these enzymes are unlikely to be regulated by Ca^2+^, although this has not yet been tested. *L. major* PPEF (*Lm*PPEF (LmjF12.0660)) is myristoylated and palmitoylated and expressed throughout the life cycle. *In vivo* the protein is localized in the endomembrane system and in the flagellar pocket. Acylation appears to be sufficient for targeting *Lm*PPEF to the flagellar pocket but not for endomembrane localization. Down-regulation of *Tb*PPEF (Tb927.1.4050) protein levels by RNAi in *T. brucei* results in a partial growth inhibition caused by the decreased level of the enzyme, but the authors suggested a total loss of PPEF activity might cause a more dramatic phenotype. The authors propose a functional, although different roles from other PPEF/RdgC molecules described in multicellular organisms.

##### 2.2.1.5 Shewanella-like (Shelps), ApaH-like (Alphs) phosphatases and kinetoplastid STPs (kSTPs)

Protein phosphatases showing similarity to bacterial phosphatase-like enzymes were described in various uni- and multicellular organisms (kinetoplasts, plants, diatoms, fungi, tunicate) suggesting these enzymes were present in common ancestor of eukaryotes, but were lost in insects, vertebrates and flowering plants amongst other taxons [47]. These protein phosphatases have an (I/L/V)D(S/T/G) motif which may have a role in altering the substrate specificity of these enzymes.

Both Alphs and Shelps are present in kinetoplastids and although the 3D structure of a *T. brucei* Alph was recently resolved, no physiological role or substrate specificity was described for this group [48].

The rest of the PPPs in this group (kSTPs) carry mutations in catalytically important regions of the enzymes, and form a large group of kinetoplastid specific phosphatases with some similarity to plant and fungal protein phosphatases. These pseudophosphatases might act as natural “substrate trapping mutants” binding to phosphosubstrates and shielding from being dephosphorylated by active phosphatases. Also while some of these pseudophosphatases lack protein phosphatase activity, they might have retained or gained other type of enzymatic activity or solely act as scaffolding molecules.

#### 2.2.2 PPM/PP2C

The PPM family of kinetoplastids show higher similarity to human and yeast PPM [8] than to their expanded plant counterparts [49]. The phosphatase activity of the PPMs depends on Mg^2+^ and Mn^2+^, and a set of 11 conserved motifs has been identified within this family [50].

From *Leishmania chagasi*, *Lc*PP2C was cloned and characterized by Burns et al. [51]. The enzyme was present in both infective promastigote and tissue amastigote stages of *L. chagasi* and *amazonensis*. Surprisingly, the enzymatic characteristics of *Lc*PP2C were remarkably similar to mammalian PP2C despite of the relative low sequence identity (30%) between these enzymes.

#### 2.2.3 FCP phosphatases

FCPs use aspartate-based catalysis to hydrolyze phosphoesther bonds and, in yeast and multicellular organisms, dephosphorylate the carboxy terminal domain (CTD) of RNA polymerase II (RNAPII). This induces interaction with TFIIF [52] and promotes recycling of RNAPII after transcription. The canonical CTD is essential for gene expression in metazoa and yeast and characterized by heptapeptide (YSPTSPS) repeats.

Many organisms, including trypanosomes, lack a canonical CTD and in these species the CTD is called a non-canonical CTD or pseudo-CTD. In kinetoplastids, despite the lack of the conserved heptad repeats of the CTD of RNAPII, phosphorylation has been detected in the so-called pseudo-CTD domain, although no CTD phosphatase was yet identified to dephosphorylate these residues [53]. There is an expansion of the FCP1 family in kinetoplastids compared to humans (Table 1), as yet, none of them was so far characterized as a CTD phosphatase.

The first phosphatase containing a DxDxT motif, characteristic to FCP phosphatases, was identified recently [54] in a substrate trapping experiment [55]. The differentiation regulator tyrosine phosphatase *Tb*PTP1 [56] was used as bait, in an attempt to find the downstream regulators of differentiation.

The identified phosphatase interacting protein with 39 kDa molecular weight (*Tb*PIP39A&B (Tb09.160.4460 and Tb09.160.4480)) was upregulated in procyclic forms, and targeted to the glycosome via a C-terminal peroxisomal targeting signal. It was found that the divalent cation dependent DxDxT phosphatase *Tb*PIP39 forms a complex with *Tb*PTP1, the latter's activity being stimulated by *Tb*PIP39, this being prevented by the differential triggers citrate/cis aconitate.

To date this is the first evidence of a signaling cascade that is directed to glycosomes (or indeed any peroxisome type organelles) and may lead to further understanding the evolution of peroxisome biogenesis and function [57].

## 3 Protein tyrosine phosphatases (PTPs)

PTPs share a common signature motif (CX_5_R) and can be classified into 4 main groups according to their catalytic domains and their substrate specificity.

The largest group in vertebrates is the cysteine (Cys) based Type I group (Fig. 2B and Table 2), which can be subdivided into Classical and Dual Specificity Phosphatases (DSPs) [12]. The classical PTPs are classified, depending on the presence or absence of transmembrane domains, into receptor or non-receptor type phosphatase groups.

The DSP groups form the largest and most diverse family of Cys based phosphatases. Members of this family dephosphorylate a wide variety of phosphosubstrates in addition to phosphoTyr and have been classified into seven subgroups [12]: MAP kinase phosphatases (MKPs), atypical DSPs, Slingshots, phosphatases of the regenerating liver (PRL), CDC14s and the members of the two classes which can dephosphorylate phospholipids-PTEN and myotubularins (Fig. 2B and Table 2).

The class II of PTPs contains the low molecular weight phosphatase (LMW PTP).

The third group of PTPs (class III) is the CDC25 family; these enzymes are both tyrosine and threonine specific, dephosphorylate cyclin dependent kinases [58], which show similarity to bacterial arsenate reductases and also have rhodanese-like domains. The rhodanese domains were first described in rhodaneses, enzymes catalyzing the transfer of a sulfane sulfur atom from thiosulfate to cyanide *in vitro*. The most relevant structural difference between rhodanese and Cdc25 enzymes is the length of the active-site loop, which in Cdc25 proteins is formed by seven residues instead of the six in sulfurtransferases; this results in a wider catalytic pocket that can accommodate a phosphorous atom, which is slightly larger than a sulfur atom [59].

The fourth PTP family comprises the EyA (eyes absent) tyrosine phosphatases, first characterized as a novel nuclear protein required for eye development in *Drosophila* and the heterogeneous family of haloacid dehalogenase (HAD) enzymes, which exhibit a wide substrate specificity dephosphorylating phospholipids, sugars, nucleotides and tyrosine or serine phosphorylated proteins and have been found only in multicellular organisms to date (reviewed in Refs. [60,61]).

### 3.1 Kinetoplastid tyrosine phosphatases

#### 3.1.1 Kinetoplastid phosphatase activities

The characterization of PTPs has lagged behind that of STPs in kinetoplastids (eleven vs. four enzymes see (Table 3)), perhaps caused by the lack of specific, permeable inhibitors against the different classes of PTPs. Until recently, vanadate and its derivatives were the only tools available to address physiological roles of tyrosine phosphatases, preventing the identification of PTP genes responsible for the measured tyrosine phosphatase activity.

Although the TriTryp kinome does not contain *bona fide* tyrosine kinases [7], tyrosine phosphorylation is detected and extent of phosphorylation differs in the tractable life cycle stages in *T. brucei* [62]. In *L. donovani* tyrosine phosphatase activity was also detected [63] suggesting that tyrosine phosphorylation occurs, though not via receptor tyrosine kinase and tyrosine kinase like kinase activities but very likely due to the activity of atypical and/or dual specific kinases.

#### 3.1.2 Membrane-bound PTP activities

Unicellular organisms, including kinetoplastids do not have any receptor type PTPs with transmembrane domains, which means that the gene encoding a life cycle specific membrane-bound protein tyrosine phosphatase activity identified in *T. brucei* [64] likely to have either a signal, which localizes the protein to membranous subcellular compartments, or be a member of a membrane bound complex. In reconstitution experiments on bloodstream form membrane proteins, 3 proteins (148, 115 and 72 kDa) exhibited PTP activity this being abolished upon vanadate treatment. No corresponding tyrosine phosphatases were present in procyclic forms.

Another membrane-bound PTP has been described in *L. major* metacyclic promastigote forms, which is translocated to the cytoplasm in promastigotes [65]. The authors raised two antibodies against the catalytic domains of the human placental PTP1B and a PTP from *T. brucei*, which cross-reacted with a 55–60 kDa protein present in the soluble detergent-extracted fraction of a *Leishmania* homogenate. In spite of the increased level of the molecule in metacyclic promastigotes compared to the procyclic forms, the specific activity of the enzyme was lower in metacyclic than in procyclic promastigotes.

#### 3.1.3 Ectophosphatases

The first a membrane bound ectophosphatase with tyrosine phosphatase activity, which is upregulated in *T. brucei* bloodstream forms was cloned by Bakalara et al. [66]. To date no physiological role was allocated to ectophosphatases, however, several hypotheses have been suggested roles in protection from cytolytic effects of extracellular ATP, dephosphorylation of ectokinase or host organism kinase substrates or involvement in signal transduction and regulating cellular adhesion (reviewed in Ref. [67]). Although some ectophosphatase can dephosphorylate tyrosine-phosphorylated substrates [67,68] they do not show any sequence similarity to PTP or STPs and for this reason they were excluded from this review.

#### 3.1.4 Cytosolic PTP activities

Bakalara et al. [69] described life cycle stage specific tyrosine phosphatase activities in *T. cruzi* and in *T. brucei*. Interestingly, the PTP activity of the lysates of the non-dividing parasites (trypomastigotes in *T. cruzi*) had a different pH optimum (pH 5.0) compared to dividing cell (epimastigotes in *T. cruzi* and bloodstream and procyclic forms in *T. brucei*) lysate, which showed the highest PTP activity at pH 7.0. The authors also showed that the tyrosine phosphatase activity in *T. brucei* procyclic forms was less than 60% of the activity measured in bloodstream forms, which according to the authors might suggest different roles for the tyrosine phosphatases in the different life cycle stages.

*In vivo* experiments in mice also showed that inhibition of PTP activities can lead to a complete block of the development of cutaneous lesions, almost complete disappearance of parasites from popliteal lymph nodes and a reduction of the liver parasite load at two weeks post-treatment.

### 3.2 Class I classical PTPs

#### 3.2.1 Non-receptor PTPs

Based on the 10 conserved motifs described in other eukaryotes [70] the kinetoplastid PTPs can be divided into three groups [8]. The members of the group 1 are *Lm*PTP1 (LmjF36.5370), and its orthologue in *T. cruzi* *Tc*PTP1 (Tc00.1047053506839.60), which show the highest similarity to vertebrate PTP1B throughout the 10 conserved domains, interestingly the *T. brucei* orthologue is missing from this group (Table 2 and Supplementary Table 2a highlighted in bold).

The second group of PTPs lacks the motif 2 (DX_2_RVXL), which is replaced by a kinetoplastid specific domain [56] and is represented in all three kinetoplastid genomes. This group comprises *Tb*PTP1 (Tb10.70.0070, which is despite of its name not an orthologue of *Lm*PTP1), *Tc*PTP2 (Tc00.1047053510187.234) and *Lm*PTP2 (LmjF36.2180).

The third group shows less similarity to the conserved motifs of mammalian PTP1B, and its members are only present in *T. brucei* and *L. major* (Supplementary Table 2a, highlighted in italic). These kinetoplastid PTPs carry substitutions in motifs 2–7 and an additional deletion between motifs 7 and 8, resulting in a PTP domain with decreased stability.

Enzymes of the first group and their role *in vivo* was investigated in *Leishmania*, where the enzyme was deleted by gene targeting, revealing that LPTP1 is necessary for survival as amastigotes in mice, but dispensable for survival as promastigotes in culture [71]. Morphologically, the *Ld*PTP1 mutant promastigotes were similar to wild type parasites. However, the *Ld*PTP1 mutants were severely attenuated in comparison to the wild type *L. donovani* with respect to survival in the liver and spleen of BALB/c mice.

The first PTP1 of group 2 was identified and characterized by our group [56]. We showed that the inactivation of the cytosolic *Tb*PTP1 in bloodstream trypanosomes by RNA interference, or a PTP1B specific inhibitor 3-(3,5-dibromo-4-hydroxy-benzoyl)-2-ethyl-benzofuran-6-sulfonicacid-(4-(thiazol-2-ylsulfamyl)-phenyl)-amide (BZ3), triggered spontaneous differentiation to procyclic forms in a subset of cells committed to differentiate. In homogeneous populations of stumpy forms, pharmacological inhibition of *Tb*PTP1 caused, cells to synchronously differentiate to procyclic forms, suggesting an important role for *Tb*PTP1 preventing differentiation to procyclic forms in the bloodstream. In an attempt to further characterize the *Tb*PTP1 signaling cascade our group recently identified an interacting partner/substrate of the *Tb*PTP1 (*Tb*PIP39) which proven to be a DxDxT phosphatase [54] and described in Section 2 of this review. While this review was in preparation, a research group published the resolved crystal structure of *Tb*PTP1 [72] and showed high structural conservation of the conventional PTP fold despite of the relatively low (24%) sequence identity to the closest *Tb*PTP1 homologue, the human PTP1. The same group also identified the nuclear RNA binding NOPP44/46 [73] as a substrate for *Tb*PTP1 in procyclic forms.

The two closest homologues of *Tb*PTP1 (Tb10.70.0070), were characterized and named by our group: *Tb*PTP2 (Tb.11.01.5450) [54,74] and *Tb*PTP3 (Tb09.v1.0350) [74]. The *Tb*PTP2 belongs to the kinetoplastid DSP group and the sequence analysis of *Tb*PTP3 revealed the closest similarity to other lipid-like dual specific phosphatases. Although *Tb*PTP1, *Tb*PTP2 and *Tb*PTP3 belong to the class I cysteine-based PTPs, we suggest different roles for these phosphatases, as ablation of each gene by RNAi resulted in different and distinct phenotypes [56,74].

#### 3.2.2 Dual specific phosphatases (DSPs)

In kinetoplastids the DSP family is the largest group of phosphatases (19/21/23 *T. brucei*/*T. cruzi*/*L. major* respectively, Supplementary Table 2b–i) containing a wide variety of phosphatases, which can be subgrouped into two groups based on domain structure and sequence homology [8]. The eukaryoticDSP (eDSPs) group, is made up of DSPs showing good conservation of classical DSP-specific domains (Supplementary Table 2b–c) [75,61], while the phosphatases in the second, atypical DSP (aDSP) group, show low similarity to eukaryote DSPs, with unusual domain organization and catalytic core (Supplementary Table 2c–g) [8].

##### 3.2.2.1 EukaryoticDSPs: phosphatases of the regenerating liver (PRL) and CDC14s

*PRL*: The PRLs are closely related to the Cdc14s and PTENs and located on various intracellular membranes. All PRLs are farnesylated, carrying a C-terminal CAAX motif, and play a role in regulating proliferation, migration and invasion of epithelial cells [76]. PRLs are not expressed in all eukaryotes and absent from the majority of protists. In kinetoplastids all the PRLs (1/4/2 in *Tb*/*Tc*/*Lm* (Supplementary Table 2b)) [8], contain the C-terminal prenylation signal, and one of these enzymes has been characterized from *T. cruzi* [77]. This enzyme, *Tc*PRL-1 (Tc00.1047053503851.24) is farnesylated in the C-terminal region, which proved to be necessary for the protein subcellular localization in the endocytic pathway of *T. cruzi*.

*CDC14s*: CDC14 phosphatases are related to the class III CDC25 phosphatases, and dephosphorylate cyclin dependent kinases (CDK), which regulate exit of mitosis and cell and centrosome division [78]. One Cdc14 orthologue was identified in each TriTryp genome (Table 2 and Supplementary Table 2c), and although none of them have been characterized, based on their important role in yeast, an essential role is likely in kinetoplastids, also.

##### 3.2.2.2 Atypical DSPs (aDSPs)

aDSPs, the most varied group of DSPs, share some characteristics of MKPs but they lack the rhodanese homology domains and can be divided into 4 groups [8].

The members of the first group (Fig. 2 and Supplementary Table 2d) carry additional domains involved in possible protein–protein interaction in addition to the DSP catalytic domain: (i) LRR-DSPs have additional Leucine Rich Repeats (LRR), the (ii) kinatases have LRR and pseudokinase domains, and finally the (iii) sole *T. cruzi* ANK-DSP has an ankyrin repeat. None of the members of this group have been characterized, but they show good homology to bacterial proteins with LRR motifs annotated as small GTP-binding proteins [8] suggesting a possible similar role in kinetoplastids, acting as scaffold proteins in signaling cascades.

The members of the second group of aDSPs are inactive and belong to the family of STYX phosphatases (Supplementary Table 2e). In other organisms these inactive enzymes are reported to act as modulators of signaling processes, via binding and controlling subcellular localization of phosphosubstrates [79].

MKPs have a conserved inactive rhodanese homology domain and can simultaneously dephosphorylate the Thr and Tyr of the TXY motif in MAPKs. Although the MKPs show little sequence homology to the classical PTPs, they share similar 3D structure around the catalytic sites of the enzymes [80]. In kinetoplastids no MKP orthologues of the human proteins were found (Table 2), but some of the kinetoplastid MKP-like phosphatases (Fig. 1 and Supplementary Table 2f), belonging to the third group of aDSPs, show similarity to plant MKPs [8] suggesting potentially conserved roles.

The lipid-like DSPs form the fourth group of aDSPs (Supplementary Table 2g); these enzymes have various mutations in the catalytic P-loop and show low homology to the classical DSP domains. Some of these enzymes show similarity to the catalytic core of the triple specific phosphatase MptpB [81] suggesting a similar type of substrate specificity for the kinetoplastid orthologues.

##### 3.2.2.3 Lipid phosphatases: phosphatase and tensin homologue deleted on chromosome 10 (PTENs) and myotubularins (MTMs)

The members of these families dephosphorylate phospholipids and act as tumor suppressors (PTEN) and regulators of endosomal trafficking (MTM) in addition to having a role in many critical signaling cascades (Supplementary Table 2h) [48,75].

PTENs were identified in the phosphatome of all three kinetoplastids, and group into two families based upon their sequence homology to other eukaryote PTENs (eukaryotic like and kinetoplastid like PTENs). Interestingly *T. cruzi* have four and *L. major* 1 eukaryotic like PTENs, while no *T. brucei* orthologue was identified. Each of the three kinetoplastids also has one kinetoplastid like PTEN enzyme (Table 2 and highlighted in bold in Supplementary Table 2h).

In vertebrates the MTMs are relatively large enzymes (from 450 to over 1000 amino acids), and they form the largest group of DSPs (16 in humans). Although in kinetoplastids the size of these enzymes are even bigger than the mammalian enzymes (from 800 to over 3000 amino acids), with long N-terminal extensions, the number of enzymes is reduced to two MTMs in each genomes, with no described functions in any of the kinetoplastids.

##### 3.2.2.4 Kinetoplastid DSP

This group contains several kinetoplastid specific DSPs (11/9/11) (Table 2 and Supplementary Table 2i), without known function. The members of this group exhibit most of the DSP motifs, but show no close homology to any of the other groups of the DSPs in other species.

##### 3.2.2.5 Slingshots

Slingshots show some similarity in their structure to DSPs, and were first identified in *Drosophila* [82]. These enzymes dephosphorylate serine phosphorylated proteins of the actin-depolymerisation factor (ADF)/cofilin group. The mammalian counterparts of the enzymes contain a 14-3-3 binding motif, a C-terminal F-actin binding site and an SH3 binding motif [83] in addition to the PTP catalytic core. In protists no obvious homologues of Slingshots were identified (Fig. 2 and Table 2).

### 3.3 Class II: low molecular weight phosphatase (LMW)/arsenate reductases (ArsC)

In vertebrates, the sole member of this family dephosphorylates tyrosine-phosphorylated substrates and is related to low molecular weight bacterial rhodanese-like phosphatases [84]. The LMW phosphatases in animals regulate cell growth, mainly by counteracting signaling from various growth factor receptors [85]. Interestingly, all the kinetoplastid LMW PTP (Supplementary Table 2j) lack their catalytic Cys residue in the active site suggesting these enzymes might be inactive.

### 3.4 Class III: cell divison cycle 25 phosphatases (Cdc25)/ARC2

Cdc25 phosphatases are abundant in all multicellular organisms, with a role of dephosphorylating and activating cyclin dependent kinases (CDK). In the TriTryp kinome, several CDK like kinases were identified, and the cell cycle has been shown to be regulated by these enzymes [86].

In *L. donovani* and *L. major* promastigotes a vanadate compound (bpV-potassium bisperoxo(1,10-phenantroline)oxovanadate V_i_) caused time and concentration dependent inhibition of phosphatase activity, resulting in an increase of cells arrested in G2/M phase of the cell cycle [87]. Upon inhibition also hyper phosphorylation of CDK1 was also observed, identifying this kinase as a possible *in vivo* substrate, as described in mammalian cells [88,89] suggesting an important role of CDC25-like phosphatases in these kinetoplastids.

Interestingly, no CDC25 homologue was found in *T. brucei* (Table 2 and Supplementary Table 2k), which might suggest that cell cycle regulation is regulated by another DSP(s) in *T. brucei*.

The CDC25-like member of this family, *Lm*ARC2 (LmjF32.2740) was described as a metalloid reductase by Zhou et al. [90] and later identified as a tyrosine phosphatase [91]. In addition to its *in vitro* phosphatase activity, the enzyme is able to reduce both As(V) and Sb(V) and is involved in the activation of pentostam, a drug containing Sb(V) used in the treatment of leishmaniasis. Mukhopadhyay et al. [92] in their recent paper resolved and characterized the 3D structure of *Lm*ARC2, and showed that the enzyme possesses a unique catalytic site, and does not belong to either the classical CDC25 group, or the As/Sb reductases but rather an enzyme with bifunctional ability to dephosphorylate phosphosubstrates and to reduce As/Sb in kinetoplastids.

## 4 Concluding remarks

The completed genome projects of the 5 kinetoplastids species made possible to assemble the kinome and phosphatome of these parasites and proved to be a powerful tool to systematically investigate the predicted functions of enzymes involved in control of protein phosphorylation–dephosphorylation.

The TriTryp phosphatome provided valuable information, showing a reduction in number of the PTPs (107 human PTPs vs. 24/30/30 *T. brucei*/*T. cruzi*/*L. major* respectively) (Table 2) which coincides with the lack of tyrosine specific protein kinases in kinetoplastids [7]. Interestingly, the increased number of atypical STPs and DSPs, in addition to the expansion of the STP family seemingly balances the total loss of receptor tyrosine phosphatases, MAPK and Slingshot families and reduced numbers of non-receptor PTPs and PPMs (Tables 2 and 3). Interestingly not expansion, but rather reduction of the phosphatome was described in *Plasmodium falciparum* according to the recently published work on the phosphatome of the human malaria parasite [93].

In addition to the high number of Kinetoplastid specific phosphatases the number of PP1s has been increased (3 human vs. 8/7/8), by gene duplication. The roles of the seemingly high number of these highly similar genes are far from fully understood.

The comparative analysis of the kinetoplastid phosphatomes shows interesting differences, which may be attributed to the different living environments of the parasites. *T. brucei*, the only extracellular parasite of the 5 kinetoplastids investigated, has an unique and smaller phosphatome compared to the intracellular *T. cruzi* and *Leishmania* ssp.

As it is shown in this review, despite of the vast amount of *in silico* data, there is still relatively little known about the *in vivo* function, substrate specificity and regulation of the kinetoplastid protein phosphatases (Table 3). To resolve this contradiction, in the future *in vivo* functional examinations need to be carried out. Combining methods of genetic manipulations, high throughput proteomics and use of specific inhibitors, with the *in silico* data should be a rational approach to further understand the regulation of signal transduction in kinetoplastids.

As the kinetoplastids phosphatase genes show low similarity to their vertebrate counterparts, targeting essential kinetoplastid protein phosphatases may be a feasible strategy to fight these pathogens, and the diseases caused by them, without interfering with the host organism signaling networks.

## Figures and Tables

**Table 1:**
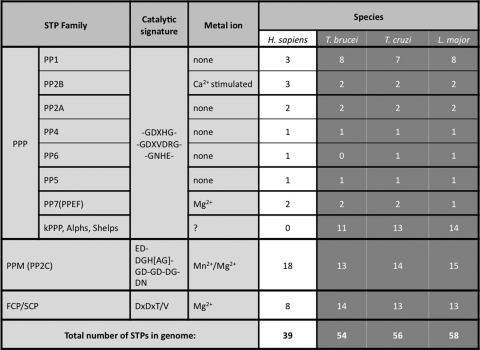
Comparison of the Ser/Thr specific protein phosphatomes of humans [94] with *T. brucei*, *T. cruzi* and *L. major*[8]. The catalytic signature motif and metal ions important for enzyme activity are shown.

**Table 2:**
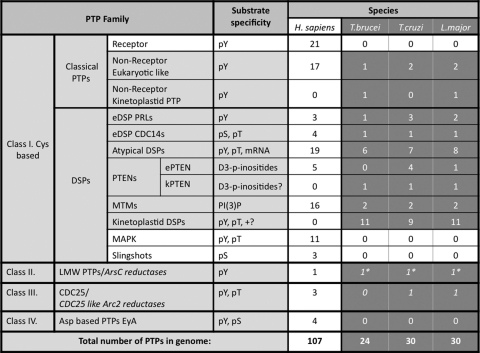
Comparison of the PTP complement [12] in humans and *T. brucei*, *T. cruzi* and *L. major*[8]. The PTP classes missing from Trypanosomatidae are highlighted in white and substrate specificity is shown.

The aDSP group includes LRR-DSPs, kinatases, ANK-DSPs, STYXs, MKP-like and lipid-like phosphatases (Fig. 1). LMW PTPs/ArsC reductases were not included in the total number of phosphatases in the kinetoplastids as they are predicted to be inactive phosphatases.

**Table 3:**
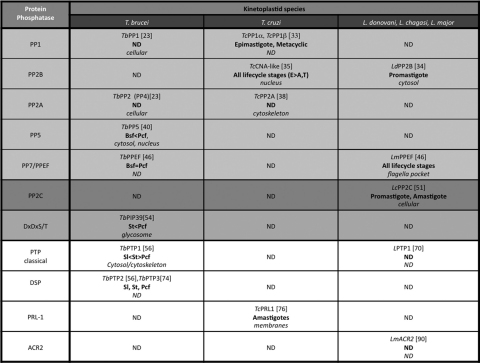
Protein phosphatases characterized in *T. brucei*, *T. cruzi* and *L. major*.

The table shows the name, the expression level in the different life cycle stages (in bold) and the subcellular localization (italic) of the characterized enzymes. *Abbreviations*: Sl, slender; St, stumpy; Pcf, procyclic form; ND, not determined.
