# Supplementary material for: Trypanosomatid protein phosphatases
Source: Mol Biochem Parasitol. 2010 Oct;173(2):53–63. doi: 10.1016/j.molbiopara.2010.05.017 (PMC2994645; doi:10.1016/j.molbiopara.2010.05.017)
Supplement: Supplementary file 1 [file mmc1.doc]

**Trypanosomatid protein phosphatases**

*Balázs Szöör

Centre for Immunity, Infection and Evolution, Institute of Immunology and Infection Research, School of Biological Sciences, University of Edinburgh, King’s Building, West Mains Road, Edinburgh EH9 3JT, UK.

*Keywords: Protein phosphatases, Trypanosomatids, Trypanosoma, Leishmania, Signal Transduction*

***Supplementary material***

Supplementary table 1

A list of identified Trypanosomatid STP sequences, according to Brenchley R et al. 2007 BMC Genomics. As a hybrid *T. cruzi* genome chosen for sequencing, it contains sequences of two closely related species. These “dupications” were listed when present in the table, and “X” denotes lack of duplicated sequence. The sequences with the systematic TriTryp gene identifiers are shown in tables based on their phylogenetic relationship: a) PP1 (the genes found in tandem gene arrays were highlighted in bold); b) PP2B/calineurin; c) PP2A; d) PP4; e) PP6; f) PP5; g) PP7/PPEF; h) kSTP/Alphs/Shelps (Alphs are highlighted in bold and Shelps were italicized); i) PPM/PP2C; j) FCP/DxDxT phosphatases.

Supplementary table 2

A list of identified Trypanosomatid STP sequences, according to Brenchley R et al. 2007 BMC Genomics. As a hybrid *T. cruzi* genome chosen for sequencing, it contains sequences of two closely related species. These “dupications” were listed when present in the table, and “X” denotes lack of duplicated sequence. The sequences with the systematic TriTryp gene identifiers are shown in tables based on their phylogenetic relationship: a) Class I., Classical PTPs (members of group 1 classical PTPs are highlighted in bold, group 2 are in normal text and group 3 are italicized); b) Class I., eukaryoticDSPs: PRLs; c) Class I., atypicalDSPs: CDC14; d) Class I., atypicalDSPs: LRR-DSP/kinatases/ANK DSP (LRR-DSPs highlighted in bold, kinatases in normal script and ANK DSPs italicized); e) Class I., atypicalDSPs: STYX; f) Class I., atypicalDSPs:MKP like; g) Class I., atypicalDSPs: Lipid like phosphatases; h) Class I., Lipid phosphatases:kPTEN/ePTEN/MTM (kPTENs highlighted in bold, ePTENs in normal script and MTMs italicized);

i) Class I., Kinetoplastid DSPs; j) Class II. LMW phosphatases/ArsC; k) Class III. CDC25/ARC2.

Supplementary table 3.

The TriTrypDB gene-identifiers and Uniprot numbers of the experimentally characterised trypanosomatid protein phosphatases described in Figure 4.
