# Supplementary material for: Trypanosomatid protein phosphatases
Source: Mol Biochem Parasitol. 2010 Oct;173(2):53–63. doi: 10.1016/j.molbiopara.2010.05.017 (PMC2994645; doi:10.1016/j.molbiopara.2010.05.017)
Supplement: Supplementary file 2 [file mmc2.doc]

**a**.

| **PP1** | | | |
| --- | --- | --- | --- |
| *T. brucei* | *T.cruzi* | *T.cruzi* duplications | *L.major* |
| Tb11.01.0450 | **Tc00.1047053506201.70** | Tc00.1047053509633.60 | LmjF15.0220 |
| Tb927.4.3560 | **Tc00.1047053506201.80** | Tc00.1047053509633.50 | LmjF28.0690 |
| **Tb927.4.3610** | Tc00.1047053509633.50 | Tc00.1047053506201.80 | LmjF31.2630 |
| **Tb927.4.3620** | **Tc00.1047053506201.30** | Tc00.1047053507671.39, Tc00.1047053507673.10 | **LmjF34.0780** |
| **Tb927.4.3630** | Tc00.1047053506315.30 | Tc00.1047053506739.130 | **LmjF34.0790** |
| **Tb927.4.3640** | Tc00.1047053507757.50 | Tc00.1047053508933.5 | **LmjF34.0800** |
| Tb927.8.7390 | Tc00.1047053508815.110 | X | **LmjF34.0810** |
| Tb927.4.5030 |  |  | **LmjF34.0850** |

**b**.

| **PP2B/calcineurin** | | | |
| --- | --- | --- | --- |
| *T. brucei* | *T. cruzi* | *T. cruzi* duplicates | *L. major* |
| Tb09.160.0480 | Tc00.1047053508413.40 | Tc00.1047053510755.138 | LmjF26.2530 |
| Tb10.70.0350 | Tc00.1047053510187.500 | X | LmjF36.1980 |

**c**.

| **PP2A** | | | |
| --- | --- | --- | --- |
| *T. brucei* | *T. cruzi* | *T. cruzi* duplicates | *L. major* |
| Tb11.01.3770 | Tc00.1047053509453.50 | Tc00.1047053511881.20 | LmjF25.1320 |
| Tb927.3.1240 | Tc00.1047053511211.60 | X | LmjF28.2670 |

**d**.

| **PP4** | | | |
| --- | --- | --- | --- |
| *T. brucei* | *T. cruzi* | *T. cruzi* duplicates | *L. major* |
| Tb11.01.8740 | Tc00.1047053511021.10 | Tc00.1047053511537.40 | LmjF32.3040 |

**e**.

| **PP6** | | | |
| --- | --- | --- | --- |
| *T. brucei* | *T. cruzi* | *T. cruzi* duplicates | *L. major* |
|  | Tc00.1047053510687.40 | X | LmjF34.4190 |

**f**.

| **PP5** | | | |
| --- | --- | --- | --- |
| *T. brucei* | *T. cruzi* | *T. cruzi* duplicates | *L. major* |
| Tb927.10.13670 (Tb10.05.0110) | Tc00.1047053507993.190 | Tc00.1047053511277.630 | LmjF18.0150 |

**g**.

| **PP7/PPEF** | | | |
| --- | --- | --- | --- |
| *T. brucei* | *T. cruzi* | *T. cruzi* duplicates | *L. major* |
| Tb927.1.4050 | Tc00.1047053506401.140 | Tc00.1047053508543.60 | LmjF12.0660 |
| Tb927.8.1130 | Tc00.1047053506529.380 | Tc00.1047053510889.80 |  |

**h**.

| kSTPs/**Alphs**/*Shelps* | | | |
| --- | --- | --- | --- |
| *T. brucei* | *T. cruzi* | *T. cruzi* duplicates | *L. major* |
| **Tb927.8.8040** | **Tc00.1047053506489.50** | X | **LmjF22.1600** |
| **Tb927.6.640** | **Tc00.1047053509211.30** | **Tc00.1047053506863.80** | **LmjF17.0580** |
| **Tb927.4.4330** | **Tc00.1047053504411.30** | **Tc00.1047053505009.30** | LmjF05.0100 |
| Tb10.70.0250 | Tc00.1047053504013.110 | Tc00.1047053508585.29, Tc00.1047053510879.10 | LmjF09.0470 |
| Tb11.01.4320 | Tc00.1047053505193.10 | Tc00.1047053506465.50 | LmjF12.0050 |
| Tb11.02.2630 | Tc00.1047053506833.30 | Tc00.1047053511731.30 | LmjF13.1510 |
| Tb927.4.1870 | Tc00.1047053507601.10 | Tc00.1047053449247.4, Tc00.1047053510351.150 | LmjF13.1570 |
| Tb927.5.4380 | Tc00.1047053507669.80 | Tc00.1047053507857.50 | LmjF22.1490 |
| Tb927.6.1230 | Tc00.1047053509591.20 | Tc00.1047053503999.60 | LmjF24.0270 |
| *Tb927.6.4630* | Tc00.1047053510187.400 | X | LmjF26.2100 |
| Tb927.6.750 | Tc00.1047053511127.400 | Tc00.1047053509029.10 | LmjF34.2770 |
|  | Tc00.1047053511825.170 | Tc00.1047053508323.190 | LmjF36.2050 |
|  | Tc00.1047053508075.14 | X | LmjF29.0440 (LMJ_0679) |
|  |  |  | LmjF30.3280 |

**i**.

| PPM/PP2C | | | |
| --- | --- | --- | --- |
| *T. brucei* | *T. cruzi* | *T. cruzi* duplicates | *L. major* |
| Tb927.7.4020 | Tc00.1047053508865.4 | Tc00.1047053420989.10, Tc00.1047053506659.39 | LmjF14.0900 |
| Tb927.5.1660 | Tc00.1047053506739.200 | Tc00.1047053506315.100 | LmjF15.0170 |
| Tb10.70.2270 | Tc00.1047053510291.30 | Tc00.1047053508659.40 | LmjF36.0530 |
| Tb11.01.6540 | Tc00.1047053506221.30 | Tc00.1047053503989.60 | LmjF32.1690 |
| Tb927.2.5050 | Tc00.1047053511625.40 | Tc00.1047053504741.30 | LmjF27.2320 (LMJ_0496) |
| Tb10.70.1410 | Tc00.1047053504163.10 | Tc00.1047053510299.79 | LmjF36.1230 |
| Tb927.6.1800 | Tc00.1047053511491.100 | X | LmjF30.0380 |
| Tb927.3.2150 | Tc00.1047053507081.50 | X | LmjF25.2060 |
| Tb927.4.3680 | Tc00.1047053504129.40 | Tc00.1047053506201.120 | LmjF06.0900 |
| Tb11.03.0390 | Tc00.1047053510303.250 | X | LmjF25.0750 |
| Tb927.4.2110 | Tc00.1047053506559.524 | Tc00.1047053506559.530 | LmjF31.1320 |
| Tb927.4.4510 | Tc00.1047053506925.150 | X | LmjF34.2500 |
| Tb11.55.0002 | Tc00.1047053509007.70 | Tc00.1047053508965.50 | LmjF34.2510 |
|  | Tc00.1047053506369.60 | Tc00.1047053510225.20 | LmjF27.1180 (LMJ_0376) |
|  |  |  | LmjF34.0730 |

**j**.

| FCP | | | |
| --- | --- | --- | --- |
| *T. brucei* | *T. cruzi* | *T. cruzi* duplicates | *L. major* |
| Tb927.2.4830 | Tc00.1047053507081.90 | Tc00.1047053503485.20 | LmjF26.0160 |
| Tb10.6k15.2490 | Tc00.1047053508181.124 | Tc00.1047053508153.840 | LmjF34.3920 |
| Tb10.70.3070 | Tc00.1047053509353.40 | Tc00.1047053430605.20 | LmjF32.0100 |
| Tb927.3.2110 | Tc00.1047053511283.120 | Tc00.1047053506525.120 | LmjF25.2030 |
| Tb09.160.4460 | Tc00.1047053511467.30 | X | LmjF24.0290 |
| Tb09.160.4480 | Tc00.1047053510747.10 | Tc00.1047053504185.30 | LmjF34.1250 |
| Tb927.3.2820 | Tc00.1047053506247.190 | X | LmjF35.2620 (LMJ_1191) |
| Tb11.02.2650 | Tc00.1047053510323.110 | Tc00.1047053508153.210 | LmjF36.6780 |
| Tb927.3.3380 | Tc00.1047053508257.210 | X | LmjF35.3520 (LMJ_1281) |
| Tb09.211.4020 | Tc00.1047053507857.70 | Tc00.1047053507669.60 | LmjF27.2180 (LMJ_0481) |
| Tb10.26.0480 | Tc00.1047053504741.200 | Tc00.1047053511621.160 | LmjF29.2400 (LMJ_0883) |
| Tb927.4.820 | Tc00.1047053504021.20 | Tc00.1047053507677.140 | LmjF35.0190 (LMJ_0953) |
| Tb10.61.2520 | Tc00.1047053506795.29 | Tc00.1047053509937.39 | LmjF03.0890 (LMJ_0246) |
| Tb927.7.3750 |  |  |  |
