# Supplementary material for: Trypanosomatid protein phosphatases
Source: Mol Biochem Parasitol. 2010 Oct;173(2):53–63. doi: 10.1016/j.molbiopara.2010.05.017 (PMC2994645; doi:10.1016/j.molbiopara.2010.05.017)
Supplement: Supplementary file 3 [file mmc3.doc]

**a**.

| Class I., Classical PTPs | | | |
| --- | --- | --- | --- |
| *T. brucei* | *T. cruzi* | *T. cruzi* duplicates | *L. major* |
|  | **Tc00.1047053506839.60** | **Tc00.1047053508717.10** | **LmjF36.5370** |
| Tb10.70.0070 | Tc00.1047053510187.234 | X | LmjF36.2180 |
| *Tb11.01.5450* |  |  | *LmjF32.0640* |

**b**.

| Class I., eukaryoticDSPs: PRL | | | |
| --- | --- | --- | --- |
| *T. brucei* | *T. cruzi* | *T. cruzi* duplicates | *L. major* |
| Tb927.8.5780 | Tc00.1047053503471.10 | Tc00.1047053506743.110, Tc00.1047053509109.120 | LmjF16.0230 |
|  | TcPRL-1 | Tc00.1047053506743.130, Tc00.1047053509109.130, Tc00.1047053503851.24 | LmjF16.0250 |
|  | Tc00.1047053510603.60 | Tc00.1047053506743.130, Tc00.1047053509109.130, Tc00.1047053503851.24 |  |

**b**.

| Class I., eukaryoticDSPs: CDC14 | | | |
| --- | --- | --- | --- |
| *T. brucei* | *T. cruzi* | *T. cruzi* duplicates | *L. major* |
| Tb11.01.4270 | Tc00.1047053503657.70 | Tc00.1047053511127.340 | LmjF09.0420 |

**d**.

| Class I., Atypical DSPs: **LRR-DSP**/ kinatases/ *ANK DSP* | | | |
| --- | --- | --- | --- |
| *T. brucei* | *T. cruzi* | *T. cruzi* duplicates | *L. major* |
| **Tb11.02.5220** | **Tc00.1047053506779.110** | **Tc00.1047053511153.90** | **LmjF28.0170** |
| Tb927.4.2460 | Tc00.1047053506559.190 | Tc00.1047053510949.10 | LmjF34.2190 |
|  | *Tc00.1047053510265.70* | *Tc00.1047053510311.40* |  |

**e.**

| Class I., Atypical DSPs: STYX | | | |
| --- | --- | --- | --- |
| *T. brucei* | *T. cruzi* | *T. cruzi* duplicates | *L. major* |
| Tb10.70.6300 | Tc00.1047053506977.10 | Tc00.1047053508637.160 | LmjF21.0700 |

**f.**

| Class I., Atypical DSPs: MKP like | | | |
| --- | --- | --- | --- |
| *T. brucei* | *T. cruzi* | *T. cruzi* duplicates | *L. major* |
| Tb927.2.4280 | Tc00.1047053509099.20 | Tc00.1047053509321.10 | LmjF27.1840 |

**g**.

| Class I., Atypical DSPs: Lipid like phosphatases | | | |
| --- | --- | --- | --- |
| *T. brucei* | *T. cruzi* | *T. cruzi* duplicates | *L. major* |
| Tb09.v1.0350 | Tc00.1047053504033.170 | Tc00.1047053510339.14 | LmjF04.0560 |
| Tb11.03.0500 | Tc00.1047053507093.330 | X | LmjF25.0570 |
|  |  |  | LmjF33.2840 |
|  |  |  | LmjF22.0250 |

**h**.

| Class I., Lipid phosphatases: **kPTEN/** ePTEN/ *MTM* | | | |
| --- | --- | --- | --- |
| *T. brucei* | *T. cruzi* | *T. cruzi* duplicates | *L. major* |
| **Tb11.03.0850** | **Tc00.1047053504109.50** | **Tc00.1047053507093.20** | **LmjF25.0230** |
|  | Tc00.1047053510099.90 | Tc00.1047053506637.100 | LmjF34.1430 |
|  | Tc00.1047053510879.160 | X |  |
|  | Tc00.1047053510943.184 | X |  |
|  | Tc00.1047053504867.114 | X |  |
| *Tb927.1.3300* | *Tc00.1047053509399.120* | *Tc00.1047053510075.30* | *LmjF20.1480* |
| *Tb927.6.870* | *Tc00.1047053510635.50* | *X* | *LmjF12.0320* |

**i**.

| Class I., Kinetoplastid DSPs | | | |
| --- | --- | --- | --- |
| *T. brucei* | *T. cruzi* | *T. cruzi* duplicates | *L. major* |
| Tb09.211.1530 | Tc00.1047053503583.80 | Tc00.1047053504625.50 | LmjF03.0510 (LMJ_0208) |
| Tb10.406.0240 | Tc00.1047053504075.6 | Tc00.1047053506933.98 | LmjF05.0220 |
| Tb11.01.4410 | Tc00.1047053504175.40 | Tc00.1047053508385.40 | LmjF08.0100 |
| Tb11.02.1640 | Tc00.1047053504741.170 | Tc00.1047053511621.190 | LmjF09.0550 |
| Tb11.02.5810 | Tc00.1047053506887.69 | Tc00.1047053510731.59 | LmjF13.0770 |
| Tb927.2.4870 | Tc00.1047053508777.60 | Tc00.1047053509029.90 | LmjF27.2210 |
| Tb927.5.3620 | Tc00.1047053509779.60 | Tc00.1047053511653.20 | LmjF28.0790 |
| Tb927.7.7160 | Tc00.1047053506737.30 | Tc00.1047053511855.60 | LmjF35.4650 (LMJ_1396) |
| Tb09.211.0210 | Tc00.1047053511725.240 | X | LmjF32.1010 |
| Tb11.01.5870 |  |  | LmjF04.0840 (L5683.01) |
| Tb10.70.3960 |  |  | LmjF05.0720 |

**j**.

| Class II. LMW phosphatases/ ArsC | | | |
| --- | --- | --- | --- |
| *T. brucei* | *T. cruzi* | *T. cruzi* duplicates | *L. major* |
| Tb09.160.2100 | Tc00.1047053504797.120 | Tc00.1047053511577.19 | LmjF01.0200 (LMJ_0020) |

**k**.

| Class III. CDC25/ARC2 | | | |
| --- | --- | --- | --- |
| *T. brucei* | *T. cruzi* | *T. cruzi* duplicates | *L. major* |
|  | Tc00.1047053508707.20 | Tc00.1047053511543.20 | LmjF32.2740 |
