# Supplementary material for: Trypanosomatid protein phosphatases
Source: Mol Biochem Parasitol. 2010 Oct;173(2):53–63. doi: 10.1016/j.molbiopara.2010.05.017 (PMC2994645; doi:10.1016/j.molbiopara.2010.05.017)
Supplement: Supplementary file 4 [file mmc4.doc]

| Gene Name | GeneDB | Uniprot | Reference |
| --- | --- | --- | --- |
| *Tb*PP1α | Tb927.8.7390 | Q57YU1 | 23. |
| *Tb*PP1β | Tb927.4.5030 | Q584K2 | 23. |
| *Tb*PP2 | Tb11.01.8740 | Q380Z0 | 23. |
| *Tc*PP1alfa | Tc00.1047053506201.70 | Q9NIV9 | 33. |
| *Tc*PP1beta | Tc00.1047053507671.39 | Q9NIV8 | 33. |
| *Tc*CnA-like | Tc00.1047053508413.40 | Q4A3I6 | 35. |
| *Tc*CnB | Tc00.1047053510519.60 | Q5V9N6 | 35. |
| *Tc*PP2A | Tc00.1047053511021.10 | **Q4D4S7** | 38. |
| *Tb*PP5 | Tb927.10.13670 (Tb10.05.0110) | Q388N2 | 40. |
| *Tb*PPEF | Tb927.1.4050 | **Q4GYH2** | 46. |
| *Lm*PPEF | LmjF12.0660 | Q4QGM7 | 46. |
| *Tb*PIP39A | Tb09.160.4460 | Q38EW0 | 54. |
| *Tb*PIP39B | Tb09.160.4480 | Q38EV9 | 54. |
| *Tb*PTP1 | Tb10.70.0070 | Q38AT7 | 56. |
| *Tb*PTP2 | Tb11.01.5450 | Q382E9 | 56. |
| *Lm*PTP1 | LmjF36.5370 | Q38EH9 | 70. |
| *Tb*PTP3 | Tb09.v1.0350 | Q38EH9 | 74. |
| *Tc*PRL1 | Tc00.1047053503851.24 | Q4CUJ8 | 76. |
| *Lm*ACR2 | LmjF32.2740 | **Q6Q1Q5** | 90. |
